# Thymosin β4 is essential for adherens junction stability and epidermal planar cell polarity

**DOI:** 10.1242/dev.193425

**Published:** 2020-12-13

**Authors:** Krishnanand Padmanabhan, Hanna Grobe, Jonathan Cohen, Arad Soffer, Adnan Mahly, Orit Adir, Ronen Zaidel-Bar, Chen Luxenburg

**Affiliations:** Department of Cell and Developmental Biology, Sackler Faculty of Medicine, Tel Aviv University, P.O. Box 39040, Tel Aviv 69978, Israel

**Keywords:** Actin cytoskeleton, Adherens junctions, Epidermis, Morphogenesis, Planar cell polarity

## Abstract

Planar cell polarity (PCP) is essential for tissue morphogenesis and homeostasis; however, the mechanisms that orchestrate the cell shape and packing dynamics required to establish PCP are poorly understood. Here, we identified a major role for the globular (G)-actin-binding protein thymosin-β4 (TMSB4X) in PCP establishment and cell adhesion in the developing epidermis. Depletion of *Tmsb4x* in mouse embryos hindered eyelid closure and hair-follicle angling owing to PCP defects. *Tmsb4x* depletion did not preclude epidermal cell adhesion *in vivo* or *in vitro*; however, it resulted in abnormal structural organization and stability of adherens junction (AJ) due to defects in filamentous (F)-actin and G-actin distribution. In cultured keratinocytes, TMSB4X depletion increased the perijunctional G/F-actin ratio and decreased G-actin incorporation into junctional actin networks, but it did not change the overall actin expression level or cellular F-actin content. A pharmacological treatment that increased the G/F-actin ratio and decreased actin polymerization mimicked the effects of *Tmsb4x* depletion on both AJs and PCP. Our results provide insights into the regulation of the actin pool and its involvement in AJ function and PCP establishment.

## Abstract (precis)

**Highlighted Article:** By regulating actin pool distribution and incorporation into junctional actin networks, thymosin β4 regulates cell–cell adhesion, planar cell polarity and epidermal morphogenesis.

## INTRODUCTION

Proper polarization of cells and cellular structures along the plane of a tissue, or planar cell polarity (PCP), is essential for embryonic development and tissue homeostasis (reviewed by Bayly and Axelrod, 2011; Eaton and Jülicher, 2011; Henderson et al., 2018; Jülicher and Eaton, 2017; McNeill, 2010). In the developing mouse epidermis, mutations in core PCP genes such as *Fzd6* and *Celsr1* prevent the alignment of hair follicles (HFs) along the anterior-posterior axis and hinder eyelid closure, a severe defect that may lead to blindness (Devenport, 2014; Devenport and Fuchs, 2008; Guo et al., 2004; Wang et al., 2016, 2006; Wang and Nathans, 2007). Although the underlying mechanisms controlling the establishment of PCP are still not completely understood, pioneering work in *Drosophila* pupal wing demonstrated that the process involves the transmission of mechanical forces and changes in cell shape and packing (Aigouy et al., 2010). Later on, similar changes in cell shape and packing were also demonstrated in the establishment of PCP in the developing mouse epidermis (Aw et al., 2016; Luxenburg et al., 2015). In *Drosophila* wing, the contraction of the wing hinge is essential for the above-mentioned cellular dynamics and PCP establishment (Aigouy et al., 2010). However, an equivalent structure does not exist in the skin and the mechanisms that orchestrate cell shape and packing dynamics to establish PCP are poorly understood.

The actin cytoskeleton and its associated adherens junctions (AJs) are major regulators of cell shape and packing. AJs are protein complexes, predominantly composed of cadherins and catenins, that occur at cell–cell junctions and play key structural and regulatory roles in epidermal development and homeostasis (Braga, 2016; Perez-Moreno et al., 2003; Rubsam et al., 2018; Sumigray and Lechler, 2015; Wickström and Niessen, 2018). AJs are linked intracellularly to the actin cytoskeleton, which is composed of filamentous (F-) actin, and numerous actin-binding proteins (Pollard, 2016). The ability of AJs to assemble and disassemble in response to intracellular and extracellular cues is crucial to their function in morphogenesis. Studies *in vitro* and *in vivo* have revealed that the assembly, stability and dynamics of AJs are all affected by junctional and perijunctional actin (Cavey et al., 2008; Hong et al., 2013). For example, a recent study using super-resolution microscopy showed that the actin cytoskeleton functions as a diffusion trap to cluster E-cadherin monomers. By regulating the cluster size, the actin cytoskeleton controls the mechanical strength of AJs (Wu et al., 2015). In line with that observation, regulators of actin polymerization, actin crosslinking and actomyosin contractility all play major roles in AJ organization and dynamics (Collinet and Lecuit, 2013). However, the involvement of G-actin-binding proteins in AJ structure and function is poorly understood.

The G-actin-binding protein thymosin-β4 (encoded by the *Tmsb4x* gene) was identified nearly 40 years ago (Low et al., 1981). TMSB4X is a small protein (43 amino acids) present in all cell types except red blood cells (Huff et al., 2001). One well-established function of TMSB4X is to sequester ATP-bound G-actin, which prevents spontaneous actin polymerization (Cassimeris et al., 1992; Sanders et al., 1992; Yu et al., 1993). More recent studies have shown that TMSB4X also influences the actin cytoskeleton by controlling the transport of G-actin between cellular compartments (Lee et al., 2013) and by supporting formin-mediated actin polymerization (Vitriol et al., 2015). Consistent with these molecular functions, TMSB4X is known to play a role in actin-based processes, such as cell migration (Sribenja et al., 2013) and extracellular matrix (ECM) remodelling (Bock-Marquette et al., 2004; Fan et al., 2009). Moreover, TMSB4X has also been implicated in the survival of cardiomyocytes, neurons and corneal epithelial cells (Philp and Kleinman, 2010) and in the regulation of gene expression in endothelial cells and breast cancer cells (Hinkel et al., 2014; Morita and Hayashi, 2013).

In the developing mouse heart, *Tmsb4x* depletion hinders cell migration and differentiation and interferes with coronary vessel development (Smart et al., 2007), whereas *Tmsb4x* depletion in mouse kidney enhances glomerular disease (Vasilopoulou et al., 2016). In cardiomyocytes, TMSB4X function is required for proper sarcomere organization (Smart et al., 2017), and in the adult mouse epidermis, TMSB4X is a positive regulator of hair growth (Gao et al., 2015). The function of TMSB4X in the skin is particularly relevant to human health, and *TMSB4X* peptides are being tested in clinical trials for the treatment of dermal ulcers and epidermolysis bullosa, a group of skin-blistering diseases (Goldstein and Kleinman, 2015; Kleinman and Sosne, 2016; Yang et al., 2019). However, the functions and underlying molecular mechanisms of TMSB4X in epidermal development have not yet been investigated.

In the present study, we investigated the roles of TMSB4X in epidermal development in the mouse. Using experiments involving genetic depletion and rescue *in utero*, together with manipulation of primary keratinocytes *in vitro*, we demonstrate that TMSB4X is crucial for PCP establishment and AJ stability. TMSB4X functions through mechanisms involving G-actin distribution and incorporation into junctional actin networks.

## RESULTS

### *Tmsb4x* depletion hinders eyelid closure but not epidermal differentiation

**Fig. 1.**
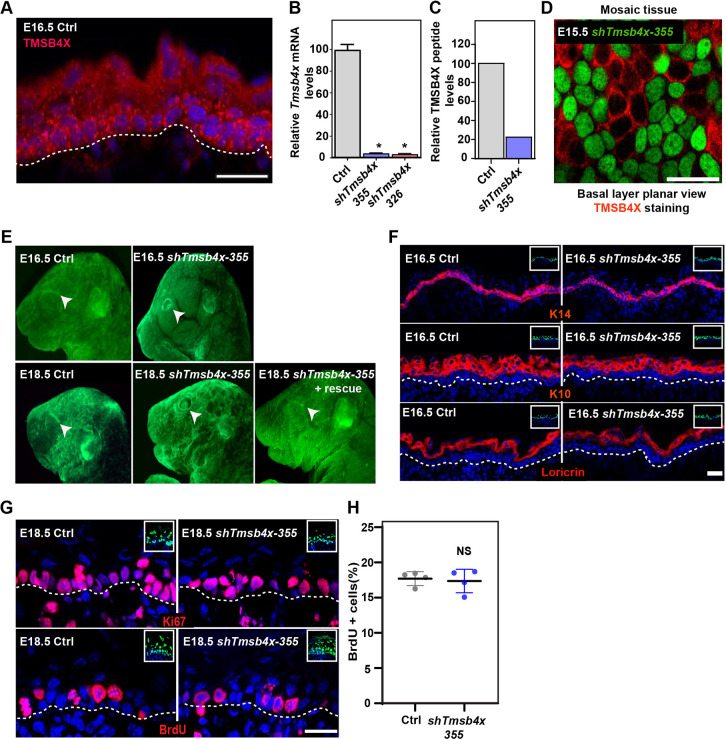
***Tmsb4x* depletion in the developing epidermis hinders eyelid closure.** (A) Sagittal views of 10-μm sections of dorsal skin from E16.5 CD1 mouse embryo immunostained for TMSB4X. (B) RT-qPCR analysis of *Tmsb4x* mRNA levels in primary mouse keratinocytes transduced with scrambled shRNA (*shScr*; Ctrl) or one of two *Tmsb4x* shRNAs (326 or 355). Data are the mean±s.d. of five experiments. *P<0.0001 for Ctrl versus *shTmsb4x-326* or *shTmsb4x-355* by unpaired two-tailed *t*-test. (C) Liquid chromatographic analysis of three peptides derived from trypsin-digested TMSB4X isolated from *shScr*- (Ctrl) and *shTmsb4x*-expressing primary mouse keratinocytes. (D) Whole-mount immunofluorescence image of dorsal skin from E15.5 CD1 embryo injected on E9 with a *Tmsb4x-355;H2B-GFP* lentivirus and immunostained for TMSB4X. (E) Stereomicroscopic images of E16.5 and E18.5 embryos infected on E9 with *shScr* (Ctrl), *shTmsb4x-355* or *shTmsb4x-355+rescue* lentiviruses. Arrowheads indicate open/closed eyes. (F) Sagittal views of 10-μm sections of dorsal skin from control (Ctrl) and *shTmsb4x-355*-transduced E16.5 embryos. Sections were immunostained for the basal layer marker K14, the differentiation marker K10 and the granular layer marker loricrin. (G) Sagittal views of 10-μm sections of dorsal skin from control and *shTmsb4x-355*-transduced E18.5 embryos. Sections were immunostained for the cell proliferation markers Ki67 and BrdU. (H) Quantification of BrdU+ cells from the data shown in E. Horizontal bars represent mean±s.d. from four embryos per condition, circles represent individual embryos. NS, not significant (*P*=0.3384) by unpaired two-tailed *t*-test. Nuclei were stained with DAPI (blue), dashed lines indicate the dermal-epidermal border, and insets show the transduced cells (H2B-GFP+). Scale bars: 20 μm.

To understand the function of TMSB4X in the developing mouse epidermis, we first examined its localization in the epidermis of E16.5 embryos by immunostaining. As expected (Huff et al., 2001), TMSB4X was detected in all the epidermal layers (Fig. 1A). Next, we screened several *Tmsb4x*-specific short hairpin RNAs (shRNAs) and identified two, *shTmsb4x-326* and *shTmsb4x-355*, that depleted *Tmsb4x* mRNA levels by 94.2±3.3% (mean±s.d.) and 95.1±2.9%, respectively, compared with control scrambled shRNA (*shScr*) in primary mouse keratinocytes (PMKs) (Fig. 1B). Mass spectrometry analysis confirmed the RT-qPCR results and demonstrated that levels of three peptides derived from TMSB4X tryptic digestion were reduced by ∼80% in PMKs expressing *shTmsb4x* compared with *shScr* (Fig. 1C).

Next, we injected the embryonic sacs of embryonic day (E) 9 wild-type mouse embryos *in utero* with lentiviruses encoding *shTmsb4x-355*, *shTmsb4x-326* or *shScr* (Ctrl) together with a GFP-tagged histone 2B reporter (H2B-GFP) to identify transduced cells (Beronja et al., 2010). Immunostaining for TMSB4X confirmed the depletion of TMSB4X in the epidermis of *shTmsb4x-355*-transduced embryos (Fig. 1D). Next, embryos were collected and inspected by fluorescence stereomicroscopy to confirm epidermal transduction (H2B−GFP+ cells) and evaluate embryo surface anatomy. Eyelid closure and fusion normally take place between days E15 and E16 in the mouse, and failure to complete these events results in open eyes at birth, potentially leading to severe corneal inflammation and blindness (Findlater et al., 1993; Heller et al., 2014). At E16.5, the eyes of control embryos were closed, whereas those of *Tmsb4x* knockdown (KD) embryos were open (Fig. 1E). To determine whether *Tmsb4x* KD merely delays, rather than prevents, eyelid closure, we also analyzed embryos that were injected with shRNA lentiviruses at E9 and examined at E18.5, several hours before delivery. Similar to the E16.5 embryos, the eyes of E18.5 *Tmsb4x* KD embryos were completely open (Fig. 1E), indicating that TMSB4X was essential for this event. Injection of a rescue virus encoding *shTmsb4x-355* and shRNA-resistant *Tmsb4x* and *H2B-GFP* (*shTmsb4x-355;H2B-GFP-P2A-Tmsb4x*) rescued the eyelid closure defect, demonstrating the specificity of our KD approach (Fig. 1E).

TMSB4X activity has been shown to play a crucial role in both the morphogenesis and differentiation of coronary vessels in the developing mouse (Smart et al., 2007). Therefore, we asked whether epidermal differentiation is affected by loss of TMSB4X function. Dorsal skin sections of *shScr-* and *shTmsb4x-355-*transduced mice were immunostained for the epidermal cell markers keratin 14 (K14, also known as Krt14; basal layer), K10 (Krt10; suprabasal layers), and loricrin (granular layer) at E16.5 (Fig. 1F) and E18.5 (Fig. S1). These analyses revealed uniform K14 staining in the basal layer, K10 staining in all suprabasal layers and loricrin staining restricted to the most apical layers in skin sections from both control and *Tmsb4x* KD embryos, indicating that TMSB4X is not required for normal epidermal differentiation in the mouse.

Because TMSB4X loss-of-function and overexpression have both been shown to affect mammalian cell proliferation (Chi et al., 2017; Wirsching et al., 2014; Xiao et al., 2014), we examined cell proliferation in the developing mouse epidermis by two methods. We first immunostained embryonic dorsal skin sections for the proliferation marker protein Ki67 (Mki67), which revealed equivalent staining patterns in control and *Tmsb4x* KD epidermis. Ki67 expression was prominent in basal layer cells and only rarely detected in suprabasal cells in both embryo types (Fig. 1G). To confirm these findings, we pulsed E18.5 pregnant mice for 2 h with the thymidine analogue 5-bromo-2′-deoxyuridine (BrdU) to label cells in S-phase. Consistent with the Ki67 staining results, we found no significant difference in the proportion of BrdU+ cells in the dorsal epidermis of control and *Tmsb4x* KD embryos (18.2±2.2% and 17.4±3.2%, respectively; Fig. 1G,H).

Taken together, these results show that TMSB4X is required for eyelid closure, an essential morphogenetic process, but not for epidermal differentiation or proliferation.

### *Tmsb4x*-depleted epidermis exhibits defects in PCP

**Fig. 2.**
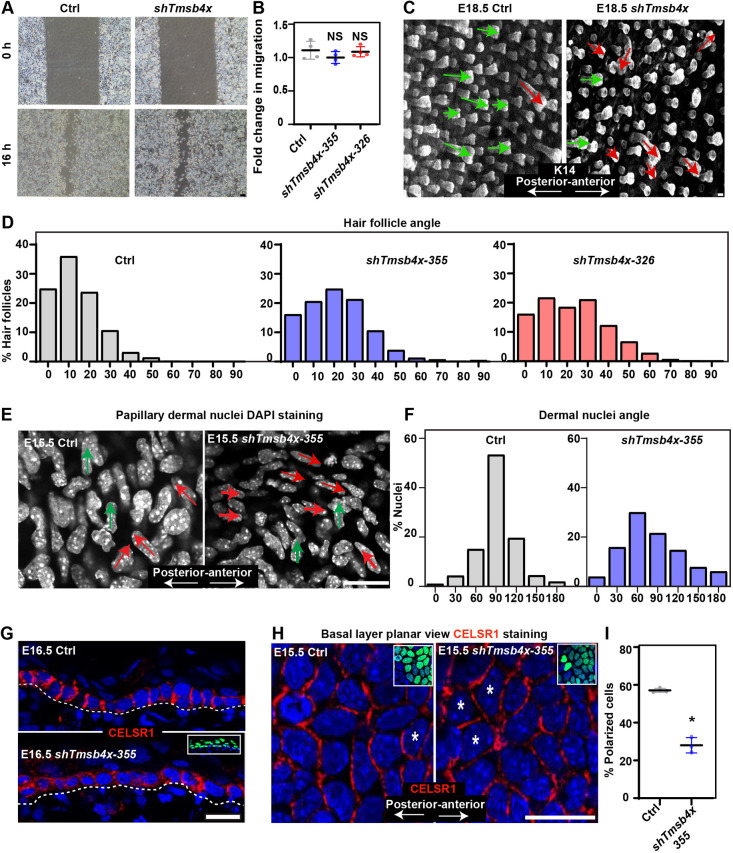
***Tmsb4x* depletion hinders planar cell polarity.** (A) Phase contrast microscopy images of *shScr*- (Ctrl) and *shTmsb4x-355-*transduced primary mouse keratinocytes in a wound-healing migration assay. Cells were incubated in medium containing 50 µM calcium. (B) Quantification of cell migration from the data shown in A. Horizontal bars represent mean±s.d. from four experiments and circles represent single experiment. NS, not significant (*P*=0.7079 and *P*=0.1719 for Ctrl versus *shTmsb4x-355* and *shTmsb4x-326*, respectively) by unpaired two-tailed *t*-test. (C) Whole-mount immunofluorescence images of control (Ctrl) and *shTmsb4x-355*-transduced E18.5 embryos immunostained for K14 (white). Green and red arrows were drawn manually to indicate normal (up to 30° relative to the anterior-posterior axis) and abnormal (more than 30° relative to the anterior-posterior axis) hair follicle angles, respectively. (D) Quantification of hair-follicle angle relative to the anterior-posterior axis from the data shown in C. *n*=442 Ctrl, 869 *shTmsb4x-355* and 465 *shTmsb4x-326* hair follicles, from three embryos per condition. (E) Whole-mount immunofluorescence images of the dorsal skin papillary dermis from control (Ctrl) and *shTmsb4x-355-*transduced E15.5 embryos. Nuclei are labelled with DAPI (white). Green and red arrows were drawn manually to indicate perpendicular and non-perpendicular dermal nuclei orientation, respectively, relative to the anterior-posterior axis. (F) Quantification of nuclei orientation from the data shown in H. *n*=667 and 510 cells from three Ctrl and *shTmsb4x-355*-transduced embryos per condition, respectively. (G) Sagittal views of 10-μm sections of dorsal skin from control (Ctrl) and *shTmsb4x-355*-transduced E16.5 embryos. Sections were immunostained for the core PCP protein CELSR1. Insets show the transduced cells (H2B-GFP+). Dashed lines indicate the dermal-epidermal border. (H) Whole-mount immunofluorescence images of dorsal skin from control (Ctrl) and *shTmsb4x-355-*transduced E15.5 embryos immunostained for CELSR1 (red) and imaged at the middle of the basal layer. Asterisks were drawn manually to indicate representative non-polarized cells. Insets show the transduced cells (H2B-GFP+). (I) Quantification of CELSR1-polarized cells from the data shown in E. Horizontal bars represent mean±s.d. and circles represent individual embryos. *n*=3 embryos per condition. *P=0.0003 by unpaired two-tailed *t*-test. Scale bars: 50 µm (A); 20 μm (C,E,G,H).

Eyelid closure is a complex process in which PCP signals orchestrate the migration of the epidermal sheet (Heller et al., 2014). Consistent with this, eyelid closure is hindered by mutations in core PCP proteins (Curtin et al., 2003; Guo et al., 2004; Wang et al., 2006; Wang and Nathans, 2007) or proteins involved in cell migration (Cohen et al., 2019; Heller et al., 2014; Huang et al., 2009). To determine whether keratinocyte migration is affected by TMSB4X loss, we performed wound-healing migration assays with PMKs transduced with *shScr* (control) or *shTmsb4x-355* in low-calcium (50 µM) medium, a condition that induces migration of keratinocytes as single cells (Zhou et al., 2013). Notably, control and *Tmsb4x* KD cells exhibited comparable migratory properties (Fig. 2A,B), indicating that TMSB4X is dispensable for the migration of individual PMKs.

In addition to orchestrating eyelid closure, PCP signals orient the angling of HFs along the anterior-posterior axis in the developing epidermis (Devenport and Fuchs, 2008; Guo et al., 2004; Luxenburg et al., 2015; Wang et al., 2006). Thus, we examined the involvement of TMSB4X in epidermal PCP by measuring HF alignment in the dorsal skin of control and *Tmsb4x* embryos at E18.5. Notably, although HFs were predominantly oriented towards the anterior of the control embryo, many HFs in *Tmsb4x* KD embryos grew laterally (Fig. 2C). Quantification of HF orientation demonstrated that ∼80% of HFs in control embryos but only ∼55% of HFs in *Tmsb4x* KD embryos were positioned within 20° of the anterior-posterior axis (Fig. 2D), supporting a role for TMSB4X in controlling PCP. Moreover, although HFs in E18.5 *Tmsb4x* KD epidermis were shorter than normal, defects in HF alignment were detected regardless of their size (Fig. S2).

PCP signals in the epidermis have been demonstrated to control cell alignment in the papillary dermis, which is the connective tissue beneath the epidermis (Aw et al., 2016). Indeed, we found that ∼55% of papillary dermal cell nuclei in control embryos were perpendicular to the anterior-posterior axis, whereas only ∼20% of the dermal cells in *Tmsb4x* KD embryos were similarly oriented (Fig. 2E,F).

To examine this process further, we determined whether loss of TMSB4X affects the localization of CELSR1, an atypical cadherin and core PCP protein. Dorsal skin sections of control embryo showed lateral localization of CELSR1 in the basal layer of E16.5 mice. However, in *shTmsb4x-355*-transduced skin, the pattern was less pronounced (Fig. 2G). To better understand CELSR1 distribution we analyzed its localization in whole-mount staining. Consistent with our previous results (Luxenburg et al., 2015), we found that 58% of basal layer cells were polarized (i.e. CELSR1 was detected only in the anterior and posterior parts of the cell) in dorsal skin from control E15.5 embryos. In contrast, fewer than 35% of basal layer cells polarized in the *Tmsb4x* KD embryos (Fig. 2H,I). Thus, TMSB4X is essential for the establishment of proper PCP in the skin.

### Loss of TMSB4X does not affect basement membrane organization, apicobasal polarity or CELSR1 mitotic internalization

Next, we investigated the mechanism by which TMSB4X regulates PCP in the epidermis. TMSB4X is an activator of integrin-linked kinase (Bock-Marquette et al., 2004), a major regulator of basement membrane (BM) assembly in the epidermis (Lorenz et al., 2007). Because BM organization, apicobasal polarity and PCP are known to be linked (Luxenburg and Geiger, 2017), we asked whether TMSB4X also affects BM organization and apicobasal polarity by immunostaining for the BM protein nidogen, the adhesion receptor integrin β4, the apicobasal polarity protein Par3, and the centrosome protein pericentrin in dorsal skin sections. This analysis indicated no difference between the staining patterns in control and *shTmsb4x-355*-transduced E16.5 embryos, with nidogen and integrin β4 detected as a thin line between the epidermis and the dermis (Fig. S3), and Par 3 and pericentrin detected in the apical part of the basal layer cells (Fig. S3). To maintain PCP in the proliferative basal layer of the epidermis, PCP proteins such as CELSR1 are internalized during mitosis and redistributed after cell division (Devenport et al., 2011). Staining of CELSR1 in dorsal skin sections showed comparable CELSR1 mitotic internalization in control and *Tmsb4x* KD embryos (Fig. S3). We also confirmed that *Tmsb4x* depletion does not affect cell proliferation in E15.5 epidermis (Fig. S4); however, we detected an increase in apoptosis in both E15.5 and E18.5 *shTmsb4x* transduced epidermis (Fig. S4). Collectively, these data indicate that the requirement for TMSB4X in PCP is not manifested through effects on BM organization, apicobasal polarity or CELSR1 mitotic internalization.

### *Tmsb4x* depletion leads to defective actin cytoskeleton and AJs in the epidermis

**Fig. 3.**
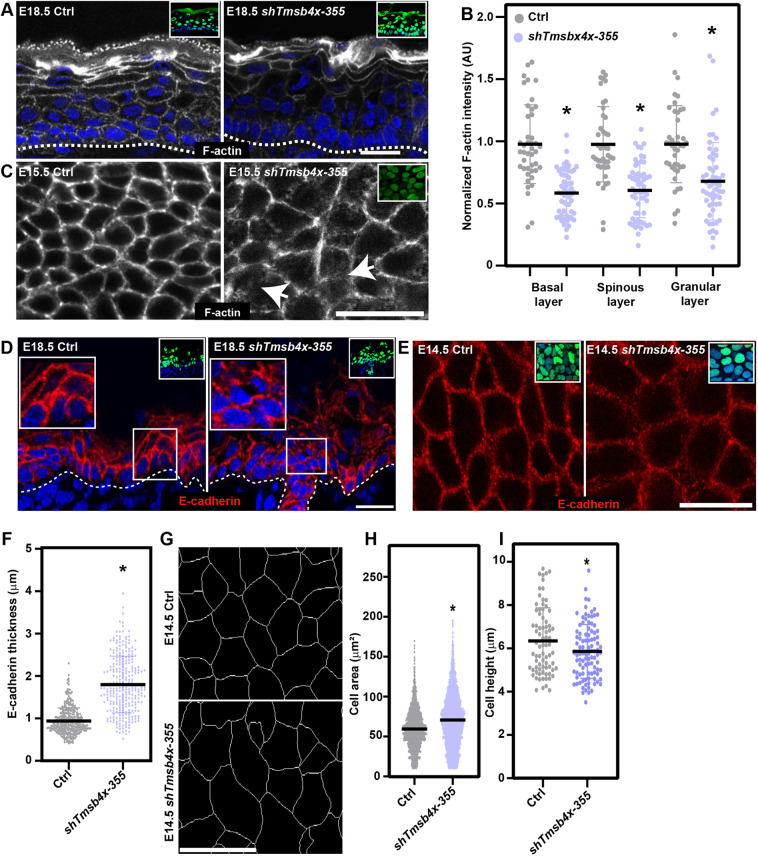
***Tmsb4x* depletion affects the actin cytoskeleton and adherens junctions.** (A) Sagittal views of dorsal skin sections from control (Ctrl) and *shTmsb4x-*transduced E18.5 embryos labelled with fluorescent Phalloidin to visualize F-actin (white). (B) Quantification of F-actin staining intensity from the data shown in A. Horizontal bars represent the mean normalized Phalloidin intensity, and circles represent individual microscopy fields. *n*=4 embryos per condition. *P*=0.0025 for Ctrl versus *shTmsb4x-355* basal layer; *P*=0.0054 for Ctrl versus *shTmsb4x-355* spinous layer; *P*=0.0128 Ctrl versus *shTmsb4x-355* granular layer by unpaired two-tailed *t*-test. (C) Whole-mount immunofluorescence images of control (Ctrl) and *shTmsb4x-355-*transduced E15.5 embryos labelled with fluorescent Phalloidin to visualize F-actin (white) and imaged at the middle of the basal layer. Arrows indicate diffuse F-actin staining. (D) Sagittal views of dorsal skin sections from control (Ctrl) and *shTmsb4x-355-*transduced E18.5 embryos immunolabelled for E-cadherin. Insets (right) show magnification of boxed areas. (E) Whole-mount immunofluorescence images of control (Ctrl) and *shTmsb4x-355-*transduced E14.5 embryos immunolabelled for E-cadherin and imaged at the middle of the basal layer. (F) Quantification of adherens junction thickness from the data shown in E. *n*=346 and 279 Ctrl and *shTmsb4x-355-*transduced cells, respectively, from three embryos per condition*.* Horizontal bars represent the mean, and circles represent individual cells. *P*<0.0001 by unpaired two-tailed *t*-test. (G) Whole-mount immunofluorescence images of control (Ctrl) and *shTmsb4x-355-*transduced E14.5 embryos immunolabelled for E-cadherin (white) with computer segmentation to visualize cell borders. (H) Quantification of cell areas from the data shown in G. *n*=1989 and 3721 Ctrl and *shTmsb4x-355-*transduced cells, respectively, from three embryos per condition*.* Horizontal bars represent the mean, and circles represent individual cells. *P*<0.0001 by unpaired two-tailed *t*-test. (I) Quantification of basal layer cell height from the data shown in D. *n*=77 and 99 Ctrl and *shTmsb4x-H2B-GFP*-transduced cells, respectively, from three embryos per condition. Horizontal bars represent the mean, and circles represent individual cells. *P*=0.0026 by unpaired two-tailed *t*-test. Nuclei were stained with DAPI (blue). Dotted lines indicate the dermal-epidermal border, and insets show the transduced cells (H2B-GFP+). Scale bars: 20 μm. *P<0.05.

We and others have shown that the actin cytoskeleton is an important regulator of PCP establishment in the mouse epidermis (Laurin et al., 2019; Luxenburg et al., 2015). To determine whether loss of TMSB4X affects the actin cytoskeleton, we first examined dorsal skin sections of E18.5 embryos labelled with the fluorescent F-actin-binding peptide Phalloidin. Although F-actin was detected predominantly at the cell cortex in both control and *Tmsb4x* KD embryos (Fig. 3A), quantification of fluorescence intensity revealed significantly lower (30-40%) F-actin levels in all epidermal layers of *Tmsb4x* KD compared with control embryos (Fig. 3B). In addition, whole-mount imaging of the middle of the basal layer showed a narrow and continuous belt of F-actin at the cell periphery in control epidermis compared with more diffuse and less continuous staining in *Tmsb4x* KD epidermis (Fig. 3C).

The actin cytoskeleton is a major regulator of AJs (Cavey et al., 2008; Hong et al., 2013; Michael and Yap, 2013; Ratheesh and Yap, 2012); therefore, we next asked whether TMSB4X is required for proper AJ organization by examining the distribution of E-cadherin in the epidermis. We found that E-cadherin was similarly enriched at the cell periphery in all epidermal layers of control and *Tmsb4x* KD embryos; however, the staining pattern was fragmented in the basal and suprabasal cells in the *Tmsb4x* KD epidermis (Fig. 3D). Whole-mount imaging of E14.5 dorsal skin, at which polarized distribution of CELSR1 can be detected for the first time (Devenport and Fuchs, 2008), showed that the average thickness of E-cadherin staining in the middle of the basal layer was ∼1 µm. However, E-cadherin staining thickness extended by ∼2-fold in *Tmsb4x* KD epidermis (Fig. 3E,F). Like AJs, tight junctions are also associated with the actin cytoskeleton; however, we detected continuous staining of the tight junction protein occludin in the apical (granular) layers of both control and *Tmsb4x* KD embryos (Fig. S5).

Defects in the actin cytoskeleton and/or AJs in the developing epidermis affect cell shape (Laurin et al., 2019; Luxenburg et al., 2015). Consistent with this, we detected significant defects in cell area and height in the epidermis of E14.5 *Tmsb4x* KD embryos compared with control embryos. Specifically, compared with the controls, *Tmsb4x* KD increased the basal layer cell area by 20% (59.5±24.4 versus 70.8±33.2 µm^2^; *P*<0.0001), and decreased the cell height by 8% (6.3±1.5 versus 5.8±1.2 µm; *P*=0.0026; Fig. 3 G-I).

Finally, we investigated the impact of the observed defects in actin and AJ distribution on intercellular adhesion and attachment to the epidermal BM using transmission electron microscopy (TEM) (Luxenburg et al., 2011). In both control and *Tmsb4x* KD embryos, we found that the membranes of neighbouring cells were sealed and desmosomes were readily detected. We also detected intact basal lamina and hemidesmosomes (Fig. S6). Taken together, these data indicate that, although the actin cytoskeleton and its associated AJs are abnormal in *Tmsb4x* KD epidermis, adhesion is maintained.

### AJ stability is perturbed upon *Tmsb4x* KD in keratinocytes

Our results thus far demonstrate that, although TMSB4X is required for PCP, its loss does not affect the key processes in PCP establishment; namely, BM organization, apicobasal polarity and CELSR1 mitotic internalization. These findings strongly suggest that the most likely cause of the observed defects in PCP in *Tmsb4x* KD epidermis lie in the aberrant actin and AJ organization. To probe this further, we turned to PMKs, which allow a more detailed analysis of actin–AJ organization and dynamics.

**Fig. 4.**
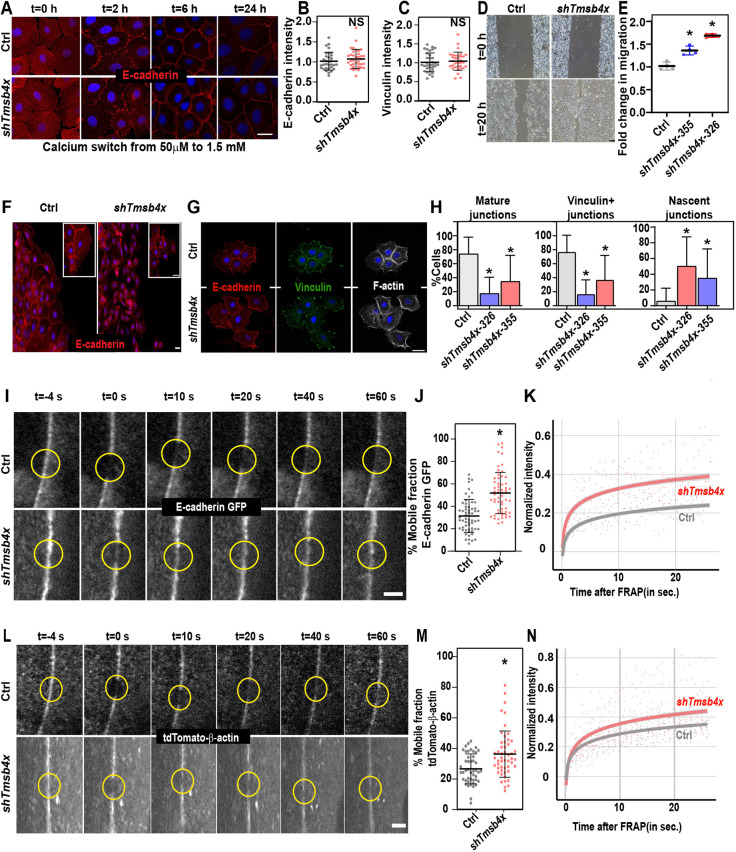
***Tmsb4x* depletion hinders adherens junction stability.** (A) *shScr*- (Ctrl) and *shTmsb4x-326*-transduced primary mouse keratinocytes (PMKs) were induced to form adherens junctions by switching from low-calcium (50 μM) to high-calcium (1.5 mM) media and then immunolabelled for E-cadherin at the indicated time points. (B,C) Quantification of E-cadherin (B) and vinculin (C) junctional intensity in *shScr*- (Ctrl) and *shTmsb4x*-transduced PMKs at 24 h after switching from low-calcium (50 μM) to high-calcium (1.5 mM) media. Horizontal bars represent mean±s.d. intensity, circles represent intensity from single microscopic fields. NS, not significant by unpaired two-tailed *t*-test. (D) Phase contrast microscopy images of *shScr-* (Ctrl) and *shTmsb4x-355*-transduced PMKs in a wound-healing migration assay. Cells were incubated in medium containing 0.3 mM calcium. (E) Quantification of cell migration from the data shown in D Horizontal bars represent mean±s.d. from four experiments and circles represent a single experiment. *P*=0.01 for Ctrl versus shTmsb4x-355- and *P*=0.0004 for Ctrl versus shTmsb4x-326-transduced cells, by unpaired two-tailed *t*-test. (F) Leading-edge PMKs from the wound-healing migration assay (D) were immunostained for E-cadherin. (G) Small colonies (4-8 cells) of *shScr-* (Ctrl) and *shTmsb4x-326*-transduced PMKs were labelled for E-cadherin, vinculin and F-actin. (H) Quantification of adherens junction organization from the data shown in G. Mean±s.d. of Ctrl, *n*=40 colonies; *shTmsb4x-355*, *n*=35 colonies; and *shTmsb4x-326*, *n*=35 colonies from three independent experiments. For nascent junctions, *P*<0.0001 for Ctrl versus *shTmsb4x-326* and *P*=0.0014 for Ctrl versus *shTmsb4x-355*. For mature junctions: *P*<0.0001 for Ctrl versus *shTmsb4x-326* and *P*=0.0003 for Ctrl versus *shTmsb4x-355*. For vinculin-positive junctions: *P*<0.0001 for Ctrl versus *shTmsb4x-326* and *P*=0.0003 for Ctrl versus *shTmsb4x-355*. All analyses were by unpaired two-tailed *t*-test. (I) Representative images of FRAP of E-cadherin-GFP in *shScr-* (Ctrl) and *shTmsb4x-326-*transduced PMKs. Yellow circles denote bleached regions. (J) Mobile fraction chart from the FRAP experiments shown in I. Ctrl, *n*=54 cells and *shTmsb4x-326*, *n*=55 cells from four independent experiments. Horizontal bar represents the mean±s.d., dots represent individual cells. *P*<0.0001 by unpaired two-tailed *t*-test. (K) Representative FRAP recovery curves from the data shown in I. (L) Representative images of FRAP of junctional tdTomato-β-actin in *shScr-* (Ctrl) and *shTmsb4x-326*-transduced PMKs. Yellow circles denote bleached regions. (M) Mobile fraction charts from the data shown in L. Ctrl, *n*=39 cells and *shTmsb4x-326*, *n*=52 cells from four independent experiments. *P*=0.0003 by unpaired two-tailed *t*-test. (N) Representative FRAP recovery curves from the data shown in L. Scale bars: 20 µm (A,F,G); 50 µm (D); 5 µm (I,L). *P<0.05.

We first ensured that our *in vivo* findings could be recapitulated *in vitro*. To examine AJ assembly, cultured PMKs were transduced with lentiviruses encoding *shScr* (control) or *shTmsb4x*-*326*, and AJ assembly was induced by increasing the medium calcium concentration from 50 µM to 1.5 mM. As observed *in vivo*, immunostaining of cells for E-cadherin identified comparable AJ formation in the control and *Tmsb4x* KD PMKs (Fig. 4A). Thus, E-cadherin could be detected in filopodia-like protrusions, also known as nascent AJs, within 2 h of the calcium switch; in a wide band at the cell periphery within 6 h, and organized into a narrow belt at the cell periphery, resembling mature AJs, within 24 h (Fig. 4A), which is consistent with previous demonstrations of the kinetics of calcium-induced AJ formation (Braga, 2016; Perez-Moreno et al., 2006; Vasioukhin et al., 2000). Notably, both the kinetics of AJ formation (Fig. 4A) and the E-cadherin and vinculin levels (Fig. 4B,C) were comparable between control and *Tmsb4x* KD PMKs, consistent with the results *in vivo*.

High extracellular calcium concentrations induce the passive assembly of AJs in densely packed PMKs; however, PCP establishment *in vivo* requires the transmission of mechanical forces and changes in cell shape and packing (Aigouy et al., 2010), which are dependent on a balance between AJ stability and remodelling. To force AJ remodelling, we examined the migration of cultured PMKs as cellular sheets using the wound-healing assay. The cells were plated at high density before switching from 50 µM- to 300 µM-calcium medium, enabling AJ formation. Migration as cellular sheets was induced by creating a gap in the monolayer (Zhou et al., 2013). Traction force microscopy has shown that, during migration, AJs at the leading edge experience high-levels of mechanical tension under these conditions (De Pascalis et al., 2018). Surprisingly, we found that *Tmsb4x* KD PMKs migrated faster than control cells in these conditions (Fig. 4D,E). Immunolabelling of E-cadherin indicated that, although control PMKs exhibited connected AJs and formed a continuous cell sheet, many *Tmsb4x* KD PMKs migrated as individual cells without intact AJs (Fig. 4F), providing an explanation for the more rapid migration of *Tmsb4x* KD compared with control PMKs (Zhou et al., 2013).

To confirm that the AJ stability defect in *Tmsb4x* KD PMKs was not specific to migrating cells, we cultured PMKs at low density before the calcium switch to induce AJ formation in small colonies (up to 10 cells). Under these conditions, AJs form only at the centre of the colony, whereas cell–ECM adhesion structures form at the perimeter of the colony and the AJs experience strong traction forces (Mertz et al., 2013). Immunolabelling of PMKs for E-cadherin together with vinculin, which is present in both cell–ECM junctions and mature AJs (Noethel et al., 2018; Sumigray et al., 2012), revealed the formation of central AJs and peripheral focal adhesions in the control PMK cultures, as expected (Fig. 4G). In contrast, although *Tmsb4x* KD PMKs clustered in close proximity, many cells did not form mature AJs (Fig. 4G)*.* To quantify junctional organization, we examined the proportion of AJs classified as nascent (E-cadherin detected at filopodia-like structures without vinculin staining) and mature (E-cadherin detected in thin junctions that also contain vinculin). Notably, nascent and mature AJs were detectable in ∼5% and 75%, respectively, of the control cells and in ∼50% and 20%, respectively, of *Tmsb4x* KD PMKs (Fig. 4H). Thus, although AJs are formed in PMKs in the absence of TMSB4X, their stability is impaired.

### Defects in AJ dynamics upon *Tmsb4x* KD in keratinocytes

AJs are dynamic structures (Aw et al., 2016; Foote et al., 2013) and their stability increases when E-cadherin and F-actin dynamics decrease (Cavey et al., 2008; Hong et al., 2013). To determine whether TMSB4X activity affects the dynamic properties of AJs and/or junctional actin, control and *Tmsb4x* KD PMKs were infected with lentiviruses encoding E-cadherin-GFP or tdTomato-β-actin, induced to form AJs, and then analyzed by fluorescence recovery after photobleaching (FRAP) to determine the AJ dynamics. E-cadherin-GFP showed recovery after photobleaching in both control and *Tmsb4x* KD PMKs (Fig. 4I). However, there was a significant (65%) increase in the proportion of mobile E-cadherin-GFP in *Tmsb4x* KD cells (31.4±14.7% versus 51.8±18.4%; Fig. 4J,K). Similarly, *Tmsb4x* depletion increased the mobile fraction of tdTomato-β-actin at the AJs compared with the control cells (26.7±9.8% versus 36.3±15.1%; Fig. 4L-N). Together, our data suggest that TMSB4X depletion does not preclude AJ formation *in vitro* or *in vivo*; however, in the absence of TMSB4X, the stability of AJs and the associated actin cytoskeleton decreases.

### Abnormal distribution of G- and F-actin in *Tmsb4x* KD cells

**Fig. 5.**
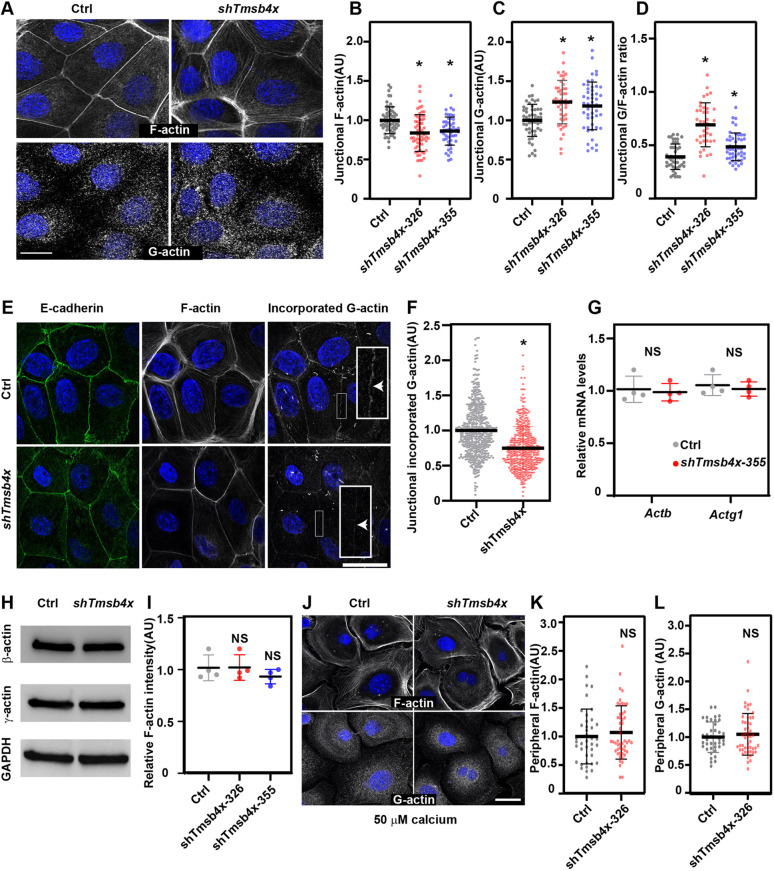
***Tmsb4x* depletion alters the junctional G/F-actin ratio in keratinocytes.** (A) *shScr-* (Ctrl) and *shTmsb4x*-transduced primary mouse keratinocytes (PMKs) were labelled for F-actin and G-actin 24 h after switching from 50 μM to 1.5 mM calcium-containing media. (B,C) Quantification of junctional F-actin (B) and G-actin (C) intensity from the data shown in A. Data are the mean±s.d. from Ctrl, *n*=59 microscopic fields; *shTmsb4x-355*, *n*=59 microscopic fields; and *shTmsb4x-326*, *n*=49 microscopic fields from four independent experiments. *P*<0.0001 for Ctrl versus *Tmsb4x-326* and versus *Tmsb4x-355* by unpaired two-tailed *t*-test. (D) Quantification of the G/F-actin ratio from the data shown in A-C. Data are the mean±s.d. *P*<0.0001 for Ctrl versus *Tmsb4x-326* and *P*=0.0064 for Ctrl versus *Tmsb4x*-355, by unpaired two-tailed *t*-test. (E) *shScr-* (Ctrl) and *shTmsb4x*-transduced PMKs were lightly permeabilized in the presence of rhodamine-actin, fixed and labelled for E-cadherin and F-actin. (F) Quantification of junctional incorporated rhodamine-actin from the data shown in E. Data are the mean±s.d. from Ctrl, *n*=490 junctions; *shTmsb4x-326*, *n*=452 junctions from three independent experiments. *P*<0.0001 by unpaired two-tailed *t*-test. (G) qPCR analysis of relative *Actb* and *Actg1* mRNA levels in *shScr-* (Ctrl) or *shTmsb4x-326-*trabnsduced PMKs. Horizontal bar represents mean±s.d. of four independent experiments and circles represent single experiments. NS, not significant. (H) Western blot analysis of Ctrl or *shTmsb4x-326-*transduced PMKs. Blots were probed with antibodies to β-actin, γ-actin or GAPDH (loading control). (I) Flow cytometry analysis of F-actin (Phalloidin) relative intensity in control, *Tmsb4x-326* and *Tmsb4x-355*-depleted PMKs. Horizontal bar represents mean±s.d. of three independent experiments and circles represent single experiments. NS, not significant. (J) Ctrl and *shTmsb4x*-326-transduced PMKs were incubated in low-calcium (50 μM) medium and labelled for F-actin and G-actin. (K,L) Quantification of peripheral F-actin (H) and G-actin (I) intensity from the data shown in G. Data are the mean±s.d. of Ctrl, *n*=68 cells and *shTmsb4x-326*, *n*=81 cells from three independent experiments. NS, not significant (*P*=0.331 and *P*=0.630 for F-actin and G-actin, respectively) by unpaired two-tailed *t*-test. Scale bars: 20µm. *P<0.05.

Because TMSB4X is an actin-binding protein, we considered that the effects of TMSB4X on AJ dynamics and stability may be mediated via regulation of the junctional actin dynamics (Braga et al., 1999; Cavey et al., 2008; Ivanov et al., 2005). In neuronal cells, TMSB4X has been shown to regulate actin dynamics by controlling G-actin distribution and the G/F-actin ratio during cell motility (Lee et al., 2013). However, it is unknown whether comparable mechanisms function in epithelial cells or, indeed, in any cell type that forms cell–cell junctions. To investigate these events and their dependence on TMSB4X in PMKs, epidermal sheets formed by incubation of control or *Tmsb4x* KD cells under high calcium conditions were labelled for G- and F-actin and E-cadherin, and junctional G- and F-actin (2 µm either side of the E-cadherin+ junction) were then quantified. F-actin was most enriched at the AJs of both control and *Tmsb4x* KD PMKs (Fig. 5A); however, the Phalloidin-labelled F-actin fluorescence intensity was significantly lower in *Tmsb4x* KD PMKs compared with control cells (1.0±0.2 versus 0.83±0.2 normalized arbitrary units; Fig. 5B). As previously reported (Cao et al., 1993; Lin, 1981), we detected diffuse and punctuated G-actin staining in both control and *Tmsb4x* KD PMKs. However, in control PMKs, G-actin was most enriched in the perinuclear region and was present at low levels in the cell periphery, adjacent to AJs, whereas G-actin was more uniformly distributed in *Tmsb4x* KD PMKs (Fig. 5A). Quantification of junctional G-actin revealed significantly higher levels at the periphery of *Tmsb4x* KD cells compared with control cells (1.0±0.2 versus 1.2±0.3 normalized arbitrary units; Fig. 5C). Based on these results, we calculated that the junctional G/F-actin ratio was significantly higher in *Tmsb4x* KD cells compared with control cells (Fig. 5D).

### Decrease in G-actin incorporation into junctional actin networks in *Tmsb4x* KD cells

Recently, Vitriol et al. showed that TMSB4X activity enhances formin-mediated F-actin assembly and elongation (Vitriol et al., 2015). To determine whether G-actin incorporation into junctional networks is regulated by TMSB4X activity, control and *Tmsb4x* KD living PMKs were lightly permeabilized, rhodamine-labelled G-actin was added, and its junctional intensity was quantified (Fig. 5E). In line with the decrease with Phalloidin-labelled junctional F-actin (Fig. 5A,B), the junctional rhodamine-labelled G-actin fluorescence intensity was significantly lower in *Tmsb4x* KD PMKs compared with control cells (1.0±0.37 versus 0.75±0.3 normalized arbitrary units; Fig. 5F).

To determine whether the above-mentioned defects in G-actin distribution and junctional incorporation were due to differential expression levels of β- and γ-actin, the two fundamental building blocks of the actin cytoskeleton, we performed qPCR and western blot analyses to measure their mRNA and protein levels, respectively. We detected comparable levels of both proteins in the control and *Tmsb4x* KD PMKs (Fig. 5G,H). Moreover, flow cytometry analysis of Phalloidin-labelled PMKs (Luxenburg et al., 2015) detected comparable levels of F-actin in control and *Tmsb4x* KD PMKs (Fig. 5I). In addition, we detected comparable peripheral G- and F-actin levels in control and *Tmsb4x* KD PMKs grown in low-calcium medium, which prevents cell–cell junction formation (Fig. 5J-L), suggesting that AJ assembly triggers the TMSB4X-mediated redistribution of G- and F-actin. Collectively, these results suggest that TMSB4X regulates AJ-dependent actin distribution and G-actin incorporation into junctional networks without affecting total β- or γ-actin levels or F-actin content.

### Latrunculin treatment mimics the effects of *Tmsb4x* depletion *in vitro* and *in vivo*

**Fig. 6.**
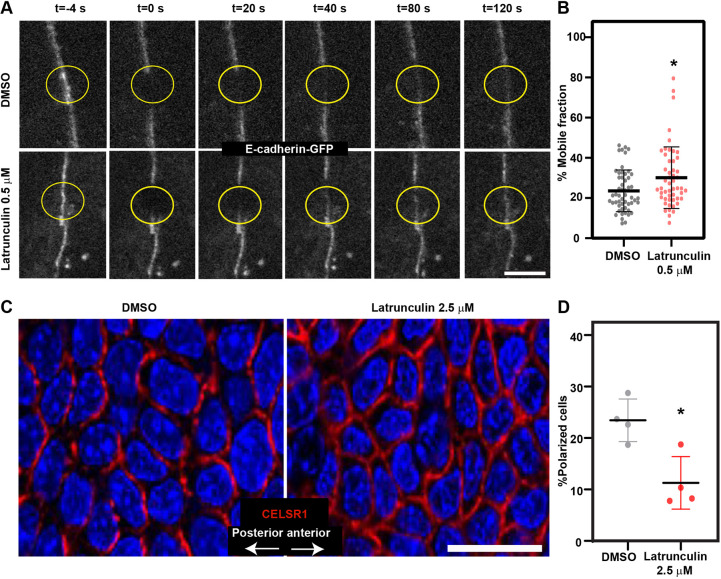
**Latrunculin treatment mimics TMSB4X loss-of-function phenotype.** (A) Representative images of FRAP of E-cadherin-GFP in PMKs treated for 30 min with DMSO (control) or 0.5 µM latrunculin. Yellow circles denote bleached regions. (B) Percentage mobile fraction from the data shown in A. DMSO, *n*=53 cells and 0.5 µM latrunculin, *n*=55 cells. *P*=0.0124 by unpaired two-tailed *t*-test. Data are mean±s.d. from three independent experiments. (C) Whole-mount immunofluorescence images of the dorsal skin from wild-type E15.5 embryos treated *ex vivo* with DMSO (control) or 2.5 µM latrunculin, and then immunostained for CELSR1 (red) and imaged at the middle of the basal layer. (D) Quantification of polarized cells from the data shown in C. Horizontal bar represents mean±s.d. from four embryos and circles represent individual embryos. *n*=4 embryos per condition. *P*=0.01 by unpaired two-tailed *t*-test. Scale bars: 5 µm (A); 20 µm (C). *P<0.05.

Finally, we asked whether the major effects of *Tmsb4x* KD on AJ dynamics and PCP establishment were mediated through modulation of the G/F-actin ratio and G-actin incorporation. To examine AJ dynamics, E-cadherin-GFP-expressing control PMKs were allowed to form epidermal sheets and then treated with vehicle (DMSO) or 0.5 µM latrunculin, a naturally occurring compound that sequesters G-actin and hinders its ability to undergo polymerization (Morton et al., 2000) and increases the cellular G/F-actin ratio (Lee et al., 2013), and the mobile fraction of E-cadherin-GFP was analyzed by FRAP. Indeed, latrunculin treatment resulted in a significant (30%) increase in the mobile fraction of E-cadherin compared with DMSO-treated cells (23.6±10.4% versus 30.1±15.3%; Fig. 6A,B).

To examine PCP, E15.5 embryos were treated *ex vivo* with DMSO or 2.5 µM latrunculin for 6 h, and dorsal skin was then stained for CELSR1 and cell polarization was quantified. CELSR1 was detected at the cell periphery of control epidermal cells, and 23.9±8.2% of the cells were polarized; however, latrunculin treatment caused a significant reduction in the proportion of polarized cells (10.5±5.0%; Fig. 6C,D), indicating that raising the G/F-actin ratio and decreasing actin polymerization was sufficient to impair epidermal PCP.

Collectively, the results presented here demonstrate an essential role for epidermal TMSB4X in AJ stability and PCP establishment. TMSB4X functions by regulating the distribution of the actin pool and the polymerization of junctional actin networks.

## DISCUSSION

PCP signals have well-established roles in regulating cell behaviour during epithelial morphogenesis, but the importance of cell shape and packing dynamics in establishing PCP has only emerged relatively recently from studies in the developing fly wing (Aigouy et al., 2010) and, even more recently, in the mouse epidermis (Aw et al., 2016; Luxenburg et al., 2015). The present study extends this work by demonstrating for the first time that the G-actin-binding protein, TMSB4X, plays a central role in cell adhesion and PCP. We show that the depletion of *Tmsb4x* in the developing epidermis gives rise to defects in the actin cytoskeleton, AJs and cell shape. It also disrupts PCP establishment and hinders the downstream processes of HF angling, dermal cell alignment and eyelid closure.

Our results demonstrate that without TMSB4X activity, junctional/cortical F-actin levels are downregulated. These results are seemingly contradictory to the notion that one of the functions of TMSB4X is to sequester G-actin and prevent spontaneous actin polymerization (Cassimeris et al., 1992; Sanders et al., 1992; Yu et al., 1993). However, they are in keeping with other roles of TMSB4X, namely regulating G-actin distribution (Lee et al., 2013) and supporting formin-mediated actin polymerization (Vitriol et al., 2015). Indeed, we found in *Tmsb4x*-depleted cultured keratinocytes that G-actin distribution is abnormal and G-actin incorporation into junctional networks was decreased. Interestingly, the deletion of TMSB4X has also been shown to disrupt F-actin-based structures in NIH323 fibroblasts (Smart and Riley, 2013) and embryonic cardiomyocytes (Smart et al., 2007). In podocytes, which exhibit a complex actin cytoskeleton, TMSB4X loss of function increases the abundance of F-actin-based stress fibres and decreases the abundance of cortical actin (Vasilopoulou et al., 2016).

Although the mechanisms by which TMSB4X regulates the actin cytoskeleton in the epidermis and other experimental systems are not entirely understood, Lee et al. showed that TMSB4X regulates actin dynamics in the growth cone of neurons and the lamellipodia of neuroblastoma cells by controlling the distribution of G-actin (Lee et al., 2013). However, in sharp contrast to these structures, which must maintain very dynamic actin to function correctly, AJ stabilization requires a decrease in junctional actin dynamics (Foote et al., 2013). We found that depletion of *Tmsb4x* in keratinocytes induced an increase in the G/F-actin ratio at junctions, and we also showed, using latrunculin, a drug that increases G/F actin ratio (Lee et al., 2013), that this defect is sufficient to explain the increase in E-cadherin mobile fraction and the decrease in AJ stability that we observed upon *Tmsb4x* depletion. These data firmly establish TMSB4X as a regulator of AJs.

TMSB4X loss of function in the mouse severely disrupts heart development (Smart et al., 2007) and promotes kidney disease (Vasilopoulou et al., 2018). PCP signals play a key role in the biology of both of these organs (Merks et al., 2018; Saburi et al., 2008) and it is tempting to speculate that a defect in PCP may explain the observed dysfunctions, at least in part. Moreover, TMSB4X-derived peptides are being tested in clinical trials and have shown promising results for the treatment of ulcers and other slow-healing wounds (reviewed by Goldstein et al., 2012; Goldstein and Kleinman, 2015; Kleinman and Sosne, 2016). Our results in the developing mouse epidermis show that TMS4BX activity is essential for PCP, which orchestrates the tissue-level behaviour of the epidermis and is essential for wound healing (Caddy et al., 2010). Additional work will be required to fully understand how TMSB4X is involved in epidermal regeneration and wound healing in adult mammals. In conclusion, our findings demonstrate a novel function for TMSB4X in G-actin distribution, actin–AJ dynamics, PCP establishment and execution of downstream processes in a complex mammalian system *in vivo*.

## MATERIALS AND METHODS

### Mice and primary mouse keratinocytes

All experimental protocols were approved by the Tel Aviv University Animal Care and Use Committee. Hsd:ICR (CD1) mice (Envigo) were used for all experiments. Epidermal keratinocytes were isolated as previously described (Nowak and Fuchs, 2009). Briefly, dorsal skin was removed from newborn mice and incubated with dispase (Sigma-Aldrich), and the epidermis was isolated and treated with trypsin (Biological Industries). Keratinocytes were plated on fibroblast feeder cells for four passages and then plated in tissue culture dishes without feeder cells.

### Lentiviruses

Lentiviruses were produced as previously described (Beronja et al., 2010; Dor-On et al., 2017). Briefly, lentiviral plasmids were generated by cloning oligonucleotides into pLKO.1-TRC (gift from David Root, Broad Institute, Cambridge, MA, USA; Addgene plasmid #10878) or LV-GFP (gift from Elaine Fuchs, Rockefeller University, New York, NY, USA; Addgene plasmid #25999) by digestion with EcoRI and AgeI, as described in the Genetic Perturbation Platform (GPP) website (http://portals.broadinstitute.org/gpp/public/resources/protocols). shRNA sequences were obtained from GPP (http://portals.broadinstitute.org/gpp/public/): *Tmsb4x* (355) construct #TRCN0000429031, target sequence 5′-AGAGGTTGGATCAAGTTTAAA-3′; *Tmsb4x*(326) construct #TRCN0000011838, target sequence 5′-CCACGAGCATTGCCTTCTTAT-3′. For the rescue virus (*shTmsb4x-355; H2B-GFP-P2A-Tmsb4x*), *shTmsb4x-355* was used for KD because it targets the 3′-untranslated region of *Tmsb4x*. *H2B-GFP* was replaced with *H2B-GFP-P2A-Tmsb4x*. For FRAP experiments, E-cadherin-GFP (Rao and Zaidel-Bar, 2016) and tdTomato-β-actin (a gift from Michael Davidson; Addgene plasmid #58068) were cloned into the control (*shScr*) or *shTmsb4x-355*-pLKO.1 plasmids digested with BamHI and KpnI.

For virus preparation, vesicular stomatitis virus G glycoprotein-pseudotyped lentiviruses were produced by transfection of 293FT cells (Invitrogen, R70007) followed by cell culture for 48-72 h. Viral supernatant was collected, filtered through a 0.45-µm filter and concentrated by ultracentrifugation using an Avanti JXN30 (Beckman Coulter) at 100,000 ***g***. Viral titres were determined by FACS analysis of infected HeLa cells.

### *In utero* lentivirus injection

Lentiviruses were injected into gestating mice as previously described (Beronja et al., 2010). Briefly, females at (E9) were anaesthetized with isoflurane and each embryo (up to six per litter) was injected with 0.4-1 µl of ∼2×10^9^ colony-forming units (CFU) of the appropriate lentiviruses. Controls were both uninfected littermates of *shTmsb4x-355/326*;*H2B-*GFP lentivirus-injected embryos and *shScr*;*H2B-GFP* lentivirus-injected embryos. The only exception is Fig. S6, in which only littermates of *shTmsb4x-355*;*H2B-*GFP lentivirus-injected embryos were used.

### *In vitro* lentivirus infection of keratinocytes

Primary mouse keratinocytes (PMKs) were generated as described above and infected as previously described (Beronja et al., 2010). Briefly, PMKs were plated at 10^5^ cells/well in six-well plates and infected with 250 µl of ∼10^7^ CFU lentiviruses (*shScr;H2B-GFP* or *shTmsb4x-355/326;H2B-GFP* with a puromycin resistance gene) in the presence of 100 µg/ml Polybrene (Sigma-Aldrich) for 48 h. Cells were the treated with 3 µg/ml puromycin (Sigma-Aldrich) for 72 h to select for infected cells. Selected cells were cultured with 1.5 µg/ml puromycin for an additional 24 h and then used in experiments.

### Semiquantitative RT-PCR

RNA was extracted from samples using the direct-zol RNA extraction kit (Zymo Research; R2060), and equal amounts of RNA were reverse transcribed using ProtoScript First Strand cDNA Synthesis Kit (New England Biolabs). Semiquantitative PCR was conducted using a StepOnePlus System (Thermo Fisher Scientific). Reactions were performed using the indicated primers and cDNA template mixed with LightCycler DNA Master SYBR Green mix and 40 cycles of amplification. The specificity of the reactions was determined by subsequent melting curve analysis. StepOnePlus software was used to adjust for background fluorescence. The number of cycles needed to reach the crossing point for each sample was used to quantify each product using the 2^-delta CT^ method. Data are presented as mRNA levels of the gene of interest normalized to peptidylprolyl isomerase B (*Ppib*) mRNA levels. The primers were: *Tmsb4x* forward 5′-AGCAGATCAGACTCTCCTCGTT-3′ and reverse 5′-GCCATATCGGGTTTGTCAGA-3′; *Actb* forward 5′-CTAAGGCCAACCGTGAAAAG-3′ and reverse 5′-ACCAGAGGCATACAGGGACA-3′; *Actg1* forward 5′-CCCAAAGCTAACAGAGAGAAGATGACG-3′ and reverse 5′-GTGGTAAAGCTGTAGCCCCGTTCA-3; *Ppib* forward 5′-GTGAGCGCTTCCCAGATGAGA-3′ and reverse 5′-TGCCGGAGTCGACAATGATG-3′.

### Liquid chromatography-mass spectrometry (LC-MS) quantification of TMSB4X peptides

Control and *Tmsb4x*-depleted PMK cells were lysed with urea and subjected to in-solution trypsin digestion followed by desalting (Gundry et al., 2009). The peptides were analyzed using nanoflow LC (nanoAcquity) coupled with high-resolution high-mass accuracy MS (Q Exactive HF). The samples were analyzed sequentially using targeted analysis mode while monitoring several theoretical TMSB4X tryptic peptides. Data were processed in two ways: using proteome Discoverer (version 2.2.0.388) and searching the UniProt mouse protein database.

### Antibodies for western blot analysis and immunofluorescence microscopy

Primary antibodies against the following proteins were purchased and used as follows: GFP (Abcam, ab13970, 1:3000), keratin 14 (K14) (BioLegend, PRB-155P, 1:1000), keratin 10 (K10) (BioLegend, PRB-159P, 1:1000), loricrin (BioLegend, Poly19051, 1:1000), nidogen (Santa Cruz Biotechnology, sc-33706, 1:2000), integrin β4 (BD Biosciences, clone 346-11A, 1:400), Ki67 (Abcam, ab15580, 1:500), BrdU (Abcam, ab6326, 1:500), CELSR1 (a gift from Elaine Fuchs, Rockefeller University, New York, NY, USA, 1:2000), Par3 (Millipore, 07-330, 1:500), pericentrin (BioLegend, PRB-432C, 1:500), E-cadherin (Cell Signaling Technology, 3195, 1:500), α-catenin (Sigma-Aldrich, C8114, 1:500), occludin (Abcam, ab31721, 1:100), vinculin (Millipore, clone 7F9, 1:200), γ-actin (Millipore, clone 2A3, 1:2000), G-actin (Millipore, clone JLA 20, 1:200), β-actin (Sigma-Aldrich, clone Ac15, 1:5000), and TMSB4X antibody (AB6019, Millipore; 1:500). Secondary antibodies were of the appropriate species/isotype reactivity conjugated to Alexa Fluor 488 or 647 or Rhodamine Red-X (Jackson ImmunoResearch, 703-545-155, 711-295-152, 712-605-153, 715-295-150, 1:500). F-actin was labelled with Phalloidin-iFluor 647 (Abcam, ab176759, 1:500). Nuclei were labelled with 4′,6-diamidino-2-phenylindole (DAPI; Sigma-Aldrich).

### Immunofluorescence and western blotting

For immunofluorescence microscopy, embryos were embedded in OCT (Scigen), frozen, sectioned at 10 μM using a Leica CM1860 cryostat, and fixed in ice-cold acetone/methanol solution or 4% formaldehyde for 10 min. Sections were then blocked with 0.3% Triton X-100, 1% bovine serum albumin, 5% normal donkey serum in phosphate-buffered saline, or in MOM Basic kit reagent (Vector Laboratories). Sections were incubated with primary antibodies (see above) overnight at 4°C and with secondary antibodies for 1 h at room temperature. For whole-mount immunofluorescence microscopy, embryos were fixed for 1-3 h in 4% formaldehyde, and the dorsal skin was removed mechanically and stained as described above.

For western blot analysis, cells were lysed with RIPA buffer (Sigma-Aldrich) and proteins were quantified using a BCA kit (Pierce). Samples of 5-20 µg protein were separated by 12% SDS-PAGE and transferred to nitrocellulose membranes. Membranes were incubated overnight at 4°C with primary antibodies to γ-actin (1:2000) and β-actin (1:5000). Blots were developed using an Enhanced Chemiluminescence Detection Kit (Biological Industries) according to the manufacturer's instructions. Images were obtain using FUSION FX7 spectra.

### Confocal microscopy

Images were acquired using a Nikon C2+ laser-scanning confocal microscope using a 60×/1.4 oil objective or a 20×/0.75 air objective (Nikon). Images were recorded as 1024×1024 square pixels. RGB images were assembled in ImageJ software (imagej.nih.gov), and panels were labelled in Adobe Illustrator CC.

### Quantification of junctional molecular composition

The fluorescence intensities of E-cadherin, vinculin, F- actin and G-actin were measured using the ImageJ FIJI package (Schindelin et al., 2012) and MorphoLibJ Plug-in (Legland et al., 2016). The E-cadherin channel was used to create a mask for measuring the intensities of the other channels. The mean grey value of the stain of interest was measured in an area 2 µm around the E-cadherin mask in a given field of view. The mean grey value of *Tmsb4x*-depleted cells was normalized to that of the control cells, and the data are presented as the normalized intensity.

### Transmission electron microscopy

A 1 cm square of dorsal skin was excised from the mouse, placed flat on a piece of paper towel and fixed in 4% paraformaldehyde and 2% glutaraldehyde in 0.05M cacodylate buffer (pH 7.4) overnight at room temperature. The tissues were then rinsed four times for 10 min each in cacodylate buffer and post-fixed and stained with 1% osmium tetroxide and 1.5% potassium ferricyanide in 0.1 M cacodylate buffer for 1 h. Tissues were then washed four times in cacodylate buffer followed by dehydration once for 10 min each in ethanol at 30%, 50%, 70%, 80%, 90% and 95% followed by three times for 20 min each in 100% anhydrous ethanol, and twice for 10 min each in propylene oxide. Following dehydration, the tissues were infiltrated with increasing concentrations of Agar 100 resin (20%, 50%, 75% and 100%) in propylene oxide for 16 h per step. The tissues were then embedded in fresh resin and allowed to polymerize in a 60°C oven for 48 h. The embedded tissue blocks were sectioned with a diamond knife on a Leica Reichert Ultracut S microtome, and ultrathin sections (80 nm) were collected onto 200 mesh, carbon/formvar-coated copper grids. The sections were then sequentially stained with uranyl acetate and lead citrate for 10 min each and viewed with a Tecnai 12 100 kV TEM (Phillips) equipped with a MegaView II CCD camera and Analysis® version 3.0 software (SoftImaging System).

### G-actin immunolabelling and quantification

G-actin was immunolabelled as previously described (Lee et al., 2013). Briefly, cells were fixed with 4% paraformaldehyde for 10 min, followed by 5 min exposure to cold acetone. To immunolabel G-actin, cells were incubated with monoclonal JLA20 anti-actin antibody (Millipore, 1:200) for 1 h at room temperature. F-actin was labelled with Phalloidin-iFluor 647 (Abcam, 1:500).

For quantification of perijunctional G- and F-actin fluorescence intensities cells were co-labelled for E-cadherin, and the E-cadherin channel was used to create a mask for measuring the intensities of the other channels. The mean grey value of the protein of interest was measured in an area 2 µm around the E-cadherin mask in a given field of view. The mean grey value of *Tmsb4x*-depleted cells was normalized to that of the control cells, and the data are presented as the normalized intensity. To calculate perijunctional G/F-actin ratio the absolute mean grey value of G-actin staining was divided by that of F-actin (Phalloidin) staining in an area 2 µm around the E-cadherin mask.

For cells grown in 50 µM calcium, the fluorescence intensities of F-actin and G-actin were measured using the ImageJ FIJI package (Schindelin et al., 2012). Cell peripheries were traced manually and a customized ImageJ macro was used to measure the intensity (mean grey value) in a 2-μm-wide band from the cell edge.

### G-actin incorporation assay

G-actin incorporation assay was performed as previously described (Kovacs et al., 2002; Verma et al., 2004). Briefly, confluent PMKs were switched to 1.5 mM calcium for 24 h. Monolayers were then incubated at 25°C for 10 min followed by live-cell permeabilization with 0.2 mg/ml Saponin (Sigma-Aldrich) in permeabilization buffer [138 mM KCl, 4 mM MgCl_2_, 20 mM HEPES (pH 7.4)] for 2 min. Then, 0.45 µM G-actin-rhodamine (Cytoskeleton, AR05) was added to the cells in permeabilization buffer for 7 min at 25°C. Cells were then fixed in 4% PFA for 10 min, followed by 5 min permeabilization with 0.2% Triton X-100 and immunolabelling for E-cadherin and F-actin.

Junctional G-actin-rhodamine incorporation was calculated in mature AJs, based on the organization of E-cadherin and F-actin. The polygon tool (ImageJ) was used to outline the E-cadherin+ mature junction and the mean grey value of the G-actin-rhodamine was measured. The mean grey value of incorporated G-actin in *Tmsb4x*-depleted cells was normalized to that of the Ctrl cells, and the data were presented as normalized intensity.

### Calcium switching and wound-healing migration assays

Control and *Tmsb4x*i-depleted PMK cells were seeded in 24-well plates in low-calcium medium (50 µM) at high confluency (8×10^4^ cells/well) or at low confluency (4×10^3^ cells/well) to examine sheet assembly or adhesion of small colonies, respectively. Upon formation of a confluent monolayer or small colonies (4 to 10 cells per colony), the medium was switched to high-calcium medium (1.5 mM) and the cells were incubated for an additional 24 h. The cells were then fixed in paraformaldehyde, and stained for E-cadherin, vinculin and F-actin. The organization/staining pattern of E-cadherin and vinculin were used to classify AJs as nascent (E-cadherin staining in filopodia-like protrusions, no vinculin staining in junctions) or mature junctions (vinculin-positive, E-cadherin and vinculin staining as a narrow band).

Migration of PMKs as individual cells or sheets was examined using wound-healing assays as previously described (Zhou et al., 2013). Briefly, control and *Tmsb4x*-depleted PMKs were plated in medium containing 50 µM or 300 µM calcium and grown to confluency. A scratch was made in the cell monolayer using a pipette tip, and wound closure was monitored over the next 24 h by capturing images at defined intervals. The migration distance was calculated by measuring the width of the scratch before (t0) and at 16 h (t16) or 24 h (t24) after incubation at 37°C. Data are presented as the fold-change in migration distance of *Tmsb4x*-depleted cells normalized to that of the control cells.

### FRAP experiments

Control and *Tmsb4x*-depleted PMK cells were transduced with E-cadherin-GFP or tdTomato-β-actin constructs and cultured in 35-mm glass-bottomed culture dishes (Eppendorf). Cells were transferred to medium containing 1.5 mM calcium for 24 h before experiments. FRAP was performed using a Nikon Ti2E microscope equipped with a Yokogawa W1 spinning disk system and a laser manipulation module (Gataca-Systems) or with a Nikon C2+ laser-scanning confocal microscope. A Plan Apo 100×/1.4 NA oil immersion objective (Nikon) was used for imaging. Cells were maintained at 37°C in a 5% CO_2_ atmosphere throughout. For the spinning disk setup, images were captured at a single *z*-plane (the apical plane of the cell) every 1 s for 4 s before photobleaching. Regions of interest were selected manually and bleached with constant area for 200 ms and subsequently imaged every 1 s for 2 min. For confocal setup, regions of interest were selected manually, and a constant circular area of 4 µm^2^ was used for photobleaching. Regions of interest were bleached with full laser power for 1 s and images were captured at a single *z*-plane (the apical plane of the cell) every 5 s for 5 min. The mobile fraction was calculated by using an ImageJ python script (https://imagej.net/Analyze_FRAP_movies_with_a_Jython_script). For the latrunculin treatment experiments, cells were treated with DMSO (0.02% final concentration) or latrunculin (0.5 µM) for 30 min before FRAP analysis.

### Quantification of PCP: CELSR1 cell polarization, HF angle and length, and dermal cell orientation

Quantification of cell polarization was performed manually as previously described (Luxenburg et al., 2015; Laurin et al., 2019). Briefly, whole-mount dorsal skin samples were immunolabelled for CELSR1 and the middle of the basal layer (∼3-4 μm above the BM) was imaged by confocal microscopy. Cells were defined as polarized when CELSR1 staining was detected only at the anterior and posterior domains.

Quantification of HF angling was performed manually as previously described (Luxenburg et al., 2015). Briefly, whole-mount dorsal skin samples were immunolabelled for K14 and imaged at low magnification in *z*-stacks from the plane of the epidermis to the tips of the HF. The images were aligned to the anterior-posterior axis of the epidermis and the HF angle was measured with the ImageJ straight line tool to draw a line between the base of the HF and its tip. The same method was also used to measure HF length.

Dermal nuclei orientation was analyzed as previously described (Aw et al., 2016). Briefly, whole-mount dorsal skin samples were labelled with DAPI and the dermal cells lying immediately beneath the basal layer were analyzed by confocal microscopy. Dermal nuclei orientation was calculated by fitting an ellipse to individual segmented nuclei using FIJI software. The orientation of each nucleus was defined by the angle at the major axis of the ellipse.

### Quantification of BrdU incorporation

Quantification of cell proliferation by BrdU incorporation was performed manually as previously described (Cohen et al., 2019). Briefly, pregnant mice were injected on E15.5 and E18.5 with 25 mg BrdU/kg body weight for 2 h, after which the embryos were collected, frozen in OCT, sectioned (10 µm) and fixed in 4% paraformaldehyde. Sections were then immersed in 4% HCl for 30 min and incubated with anti-BrdU primary antibody (Abcam, 1:200). To quantify cell proliferation, sections were imaged by confocal microscopy and the percentage GFP+ BrdU+ double-positive cells among total GFP+ cells was calculated.

### Quantification of F-actin intensity

Dorsal skin sections were incubated with Alexa Fluor 647-conjugated Phalloidin overnight at 4°C, and samples were imaged using confocal microscopy with a 60×/1.4 objective that generated optical sections of 0.49 µm. Fields in which more than 90% of the epidermal cells were transduced (GFP+) were included in the quantification. In each field, basal, spinous and granular layers were manually segmented, and their mean grey value was calculated using ImageJ.

### Quantification of F-actin content

Total F-actin levels were measured as previously described (Luxenburg et al., 2015). Briefly, Ctrl and Tmsb4x-depleted confluent PMKs were switched to 1.5 mM calcium for 24 h. Monolayers were then trypsinized, fixed and labelled with Phalloidin-iFluor 647 (Abcam, 1:500) and analyzed in cytoFLEX flow cytometer (Beckman). The median F-actin fluorescence intensity in Tmsb4x-depleted cells was normalized to that of Ctrl cells.

### Quantification of cell shape

To quantify the basal layer cell shape, whole-mount samples were labelled for E-cadherin and confocal images were collected at a single plane through the middle of the basal layer. Images were then filtered using a two-dimensional band-pass filter, and cells were segmented based on E-cadherin staining using a watershed algorithm. Measurement of cell area was performed using packing analyzer software v2 (Aigouy et al., 2010). To measure cell height, 10 µm sagittal sections of dorsal skin were stained for E-cadherin and imaged with confocal microscopy. Cell height was measured manually by drawing a line from the basal to the apical part of the cell using the line tool in ImageJ.

### Latrunculin treatment

Embryos were collected on E15.5 and incubated with 2.5 μM latrunculin or an equal final concentration of DMSO for 6 h in serum-free DMEM (Biological Industries). Following treatment, embryos were fixed, stained and analyzed for CELSR1 as described above.

### Statistical analysis

Quantitative data are shown as the mean±s.d. Analyses were performed using Prism (GraphPad). Sample sizes and the specific tests performed are indicated in the figure legends. No statistical method was used to predetermine the sample size. Experiments were not randomized; investigators were not blinded to allocation during experiments and outcome assessments.

## Supplementary Material

Supplementary information
