# Supplementary material for: Thymosin β4 is essential for adherens junction stability and epidermal planar cell polarity
Source: Development. 2020 Dec 13;147(23):dev193425. doi: 10.1242/dev.193425 (PMC7758630; doi:10.1242/dev.193425)
Supplement: Supplementary information [file develop-147-193425-s1.pdf]

Fig. S1

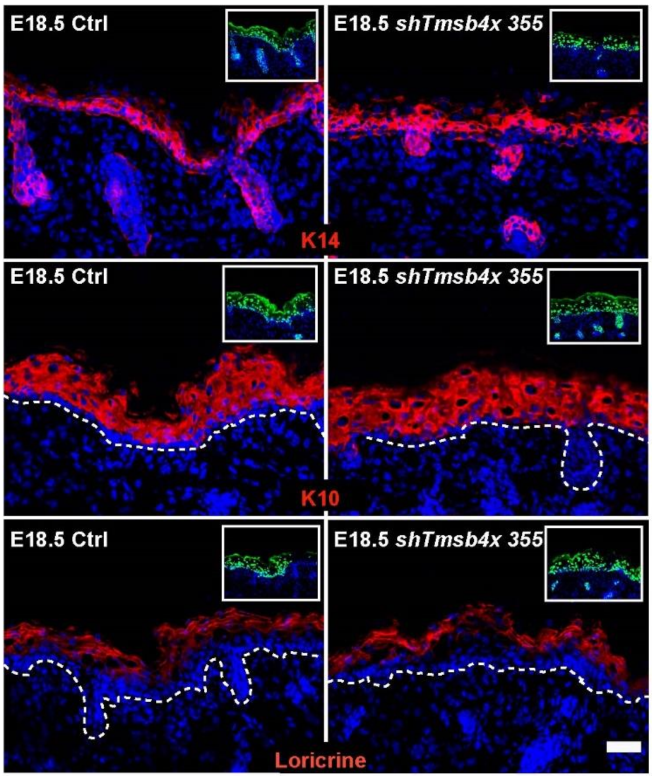

Fig. S1. Epidermal differentiation is normal in *Tmsb4x*-depleted embryos.

Sagittal views of 10-µm dorsal skin sections from control (Ctrl) and *shTmsb4x-355*-transduced E18.5 embryos. Sections were immunostained for the basal layer marker K14, the differentiation marker K10, and the granular layer marker loricrin. Nuclei were stained with DAPI. Dotted lines indicate the dermal–epidermal border, and insets show the transduced cells (H2B-GFP+). Scale bar = 20 µm.

Fig. S2

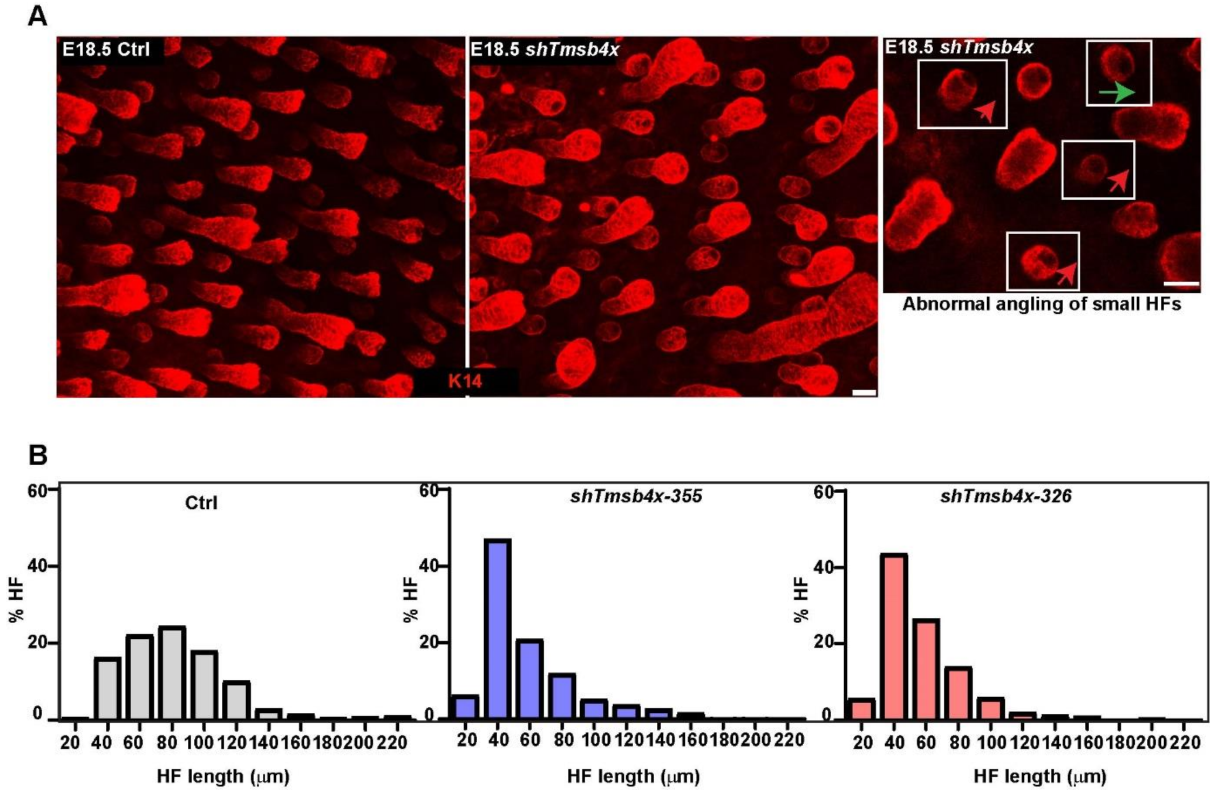

Fig. S2. *Tmsb4x* depletion hinders hair follicle growth.

(A) Whole-mount immunofluorescence images of control (Ctrl) and *shTmsb4x-355*-transduced E18.5 embryos immunostained for K14 (red). Framed HF denotes short HFs with normal or abnormal angling (green and red arrows respectively)

(B) Quantification of hair-follicle length from the data shown in (A). n = 442, control and 869 *Tmsb4x-355*-hair follicles, from three embryos per condition.

Fig. S3

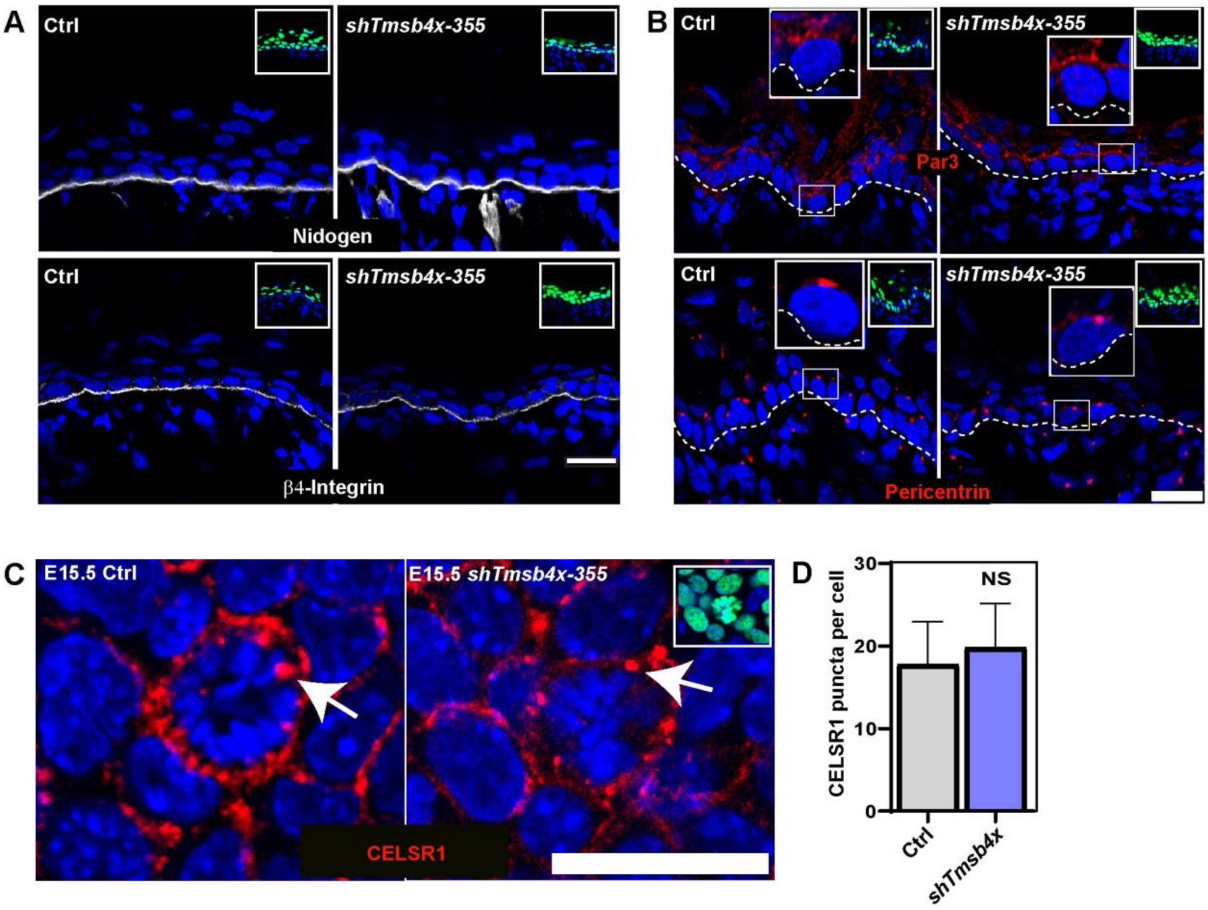

Fig. S3. TMSB4X does not affect basement membrane organization, apicobasal polarity, or mitotic internalization of CELSR1.

(A) Sagittal views of dorsal skin sections from E16.5 control (Ctrl) and sh*Tmsb4x-355*-transduced embryos immunostained for the basement membrane markers nidogen and β4 integrin.

(B) Sagittal views of dorsal skin sections from E16.5 Ctrl and *shTmsb4x-355*-transduced embryos immunostained for the polarity protein Par3 and the centrosome marker pericentrin. Upper left insets show 2× digital magnifications of the lower boxed areas.

(C) Whole-mount immunofluorescence of E15.5 Ctrl and sh*Tmsb4x-355*-transduced embryos immunostained for CELSR1. Images reveal no obvious defects in the mitotic internalization of CELSR1. Arrows indicate mitotic cells.

(D) quantification of CELSR1 puncta in mitotic cells from the data shown in (C). Data are the mean ± SD from Ctrl, n = 16 cells; *shTmsb4x-355,* n = 20 cells from three embryos . NS, not significant (P=0.3972) by unpaired two tailed t test

Nuclei were stained with DAPI (blue). Upper right insets show the transduced cells (H2B-GFP+). Scale bars = 20 µm.

Development • Supplementary information

Fig. S4

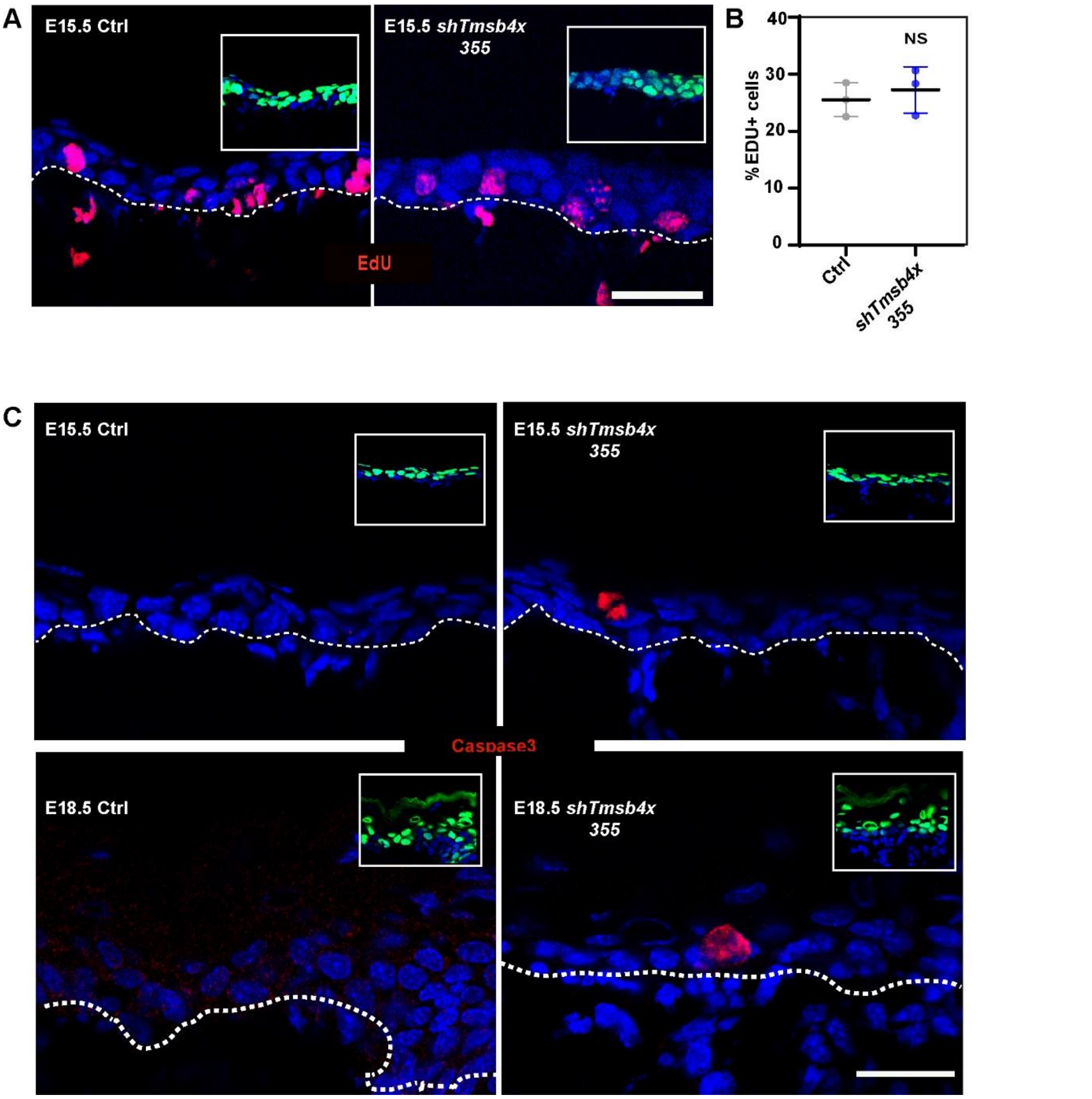

Fig. S4. *Tmsb4x*-depleted epidermis exhibits normal cell proliferation but an increase in cell death.

(A) Sagittal views of 10-µm sections of dorsal skin from control and *shTmsb4x-355*-transduced E15.5 embryos. Sections were labeled for EdU.

(B) Quantification of EdU+ cells from the data shown in (A). Horizontal bar represents mean ± SD from three embryos per condition and circles represent individual experiment.NS, not significant (P = 0.9876) by unpaired two tailed t test.

(C) Sagittal views of 10-µm sections of dorsal skin from control and *shTmsb4x-355*-transduced E15.5 and E18.5 embryos. Sections were labeled for activated caspase 3.

Nuclei were stained with DAPI (blue), dotted lines indicate the dermal–epidermal border, and insets show the transduced cells (H2B-GFP+). All scale bars = 20 µm.

Development • Supplementary information

Fig. S5

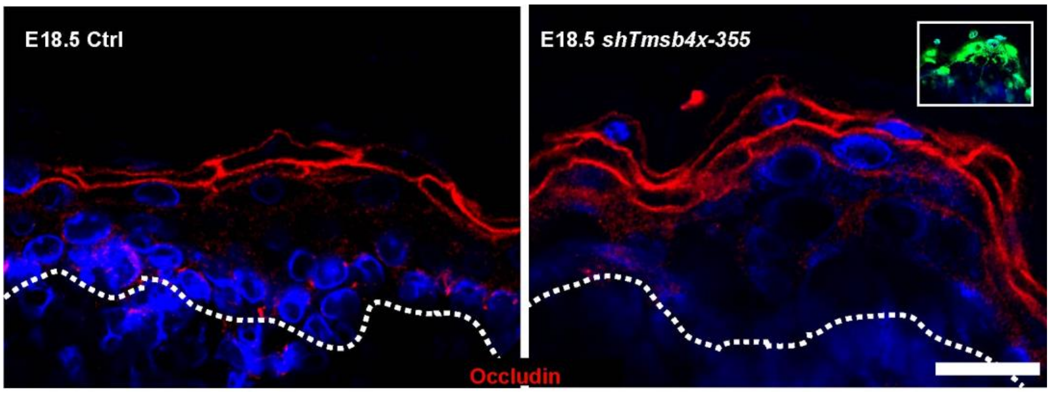

Fig. S5. Normal distribution of occludin in *Tmsb4x*-depleted epidermis.

Sagittal views of 10-µm sections of dorsal skin from control (Ctrl) and *shTmsb4x-355*-transduced E18.5 embryos. Sections were immunostained for the tight junction protein occludin. Nuclei were stained with DAPI. Dotted lines indicate the dermal–epidermal border, and the inset shows the transduced cells (H2B-GFP+). Scale bar = 20 µm.

Fig. S6

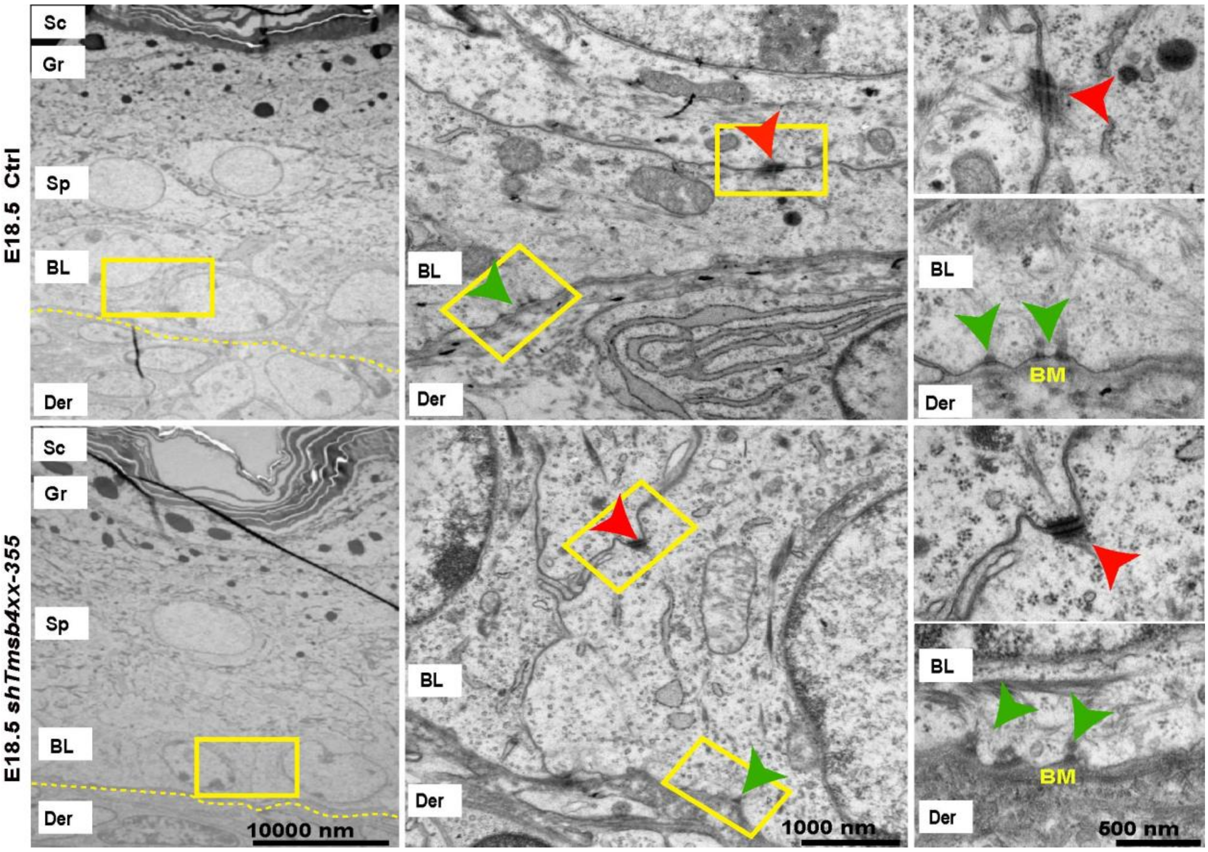

Fig. S6. Adhesion is maintained in *Tmsb4x*-depleted epidermis.

Transmission electron microscopic images of epidermis wild-type and *shTmsb4x-355*-transduced E18.5 embryos. Yellow-boxed areas in the left column are shown at higher magnifications in the middle and right columns. Red and green arrowheads indicate desmosomes and hemidesmosomes, respectively. Scale bars = 10,000, 1000, and 500 nm for the left, middle, and right panels, respectively. Der, dermis; BL, basal layer; SP, spinous layer; Gr, granular layer; Sc, stratum corneum.
